# Weight-Independent Mechanisms of Glucose Control After Roux-en-Y Gastric Bypass

**DOI:** 10.3389/fendo.2018.00530

**Published:** 2018-09-10

**Authors:** Blandine Laferrère, François Pattou

**Affiliations:** ^1^Division of Endocrinology, New York Obesity Nutrition Research Center, Department of Medicine, Columbia University College of Physicians and Surgeons, New York, NY, United States; ^2^Translational Research on Diabetes, UMR 1190, Inserm, Université Lille, Lille, France; ^3^Endocrine and Metabolic Surgery, CHU Lille, Lille, France

**Keywords:** gastric bypass surgery, beta cell function, glucagon-like peptide 1 (GLP-1), bile acids, microbiome, sodium glucose transporter 1 (SGLt1), type 2 diabetes

## Abstract

Roux-en-Y gastric bypass results in large and sustained weight loss and resolution of type 2 diabetes in 60% of cases at 1–2 years. In addition to calorie restriction and weight loss, various gastro-intestinal mediated mechanisms, independent of weight loss, also contribute to glucose control. The anatomical re-arrangement of the small intestine after gastric bypass results in accelerated nutrient transit, enhances the release of post-prandial gut hormones incretins and of insulin, alters the metabolism and the entero-hepatic cycle of bile acids, modifies intestinal glucose uptake and metabolism, and alters the composition and function of the microbiome. The amelioration of beta cell function after gastric bypass in individuals with type 2 diabetes requires enteric stimulation. However, beta cell function in response to intravenous glucose stimulus remains severely impaired, even in individuals in full clinical diabetes remission. The permanent impairment of the beta cell may explain diabetes relapse years after surgery.

The prevalence of severe obesity, defines as body mass index (BMI) above 40 kg/m^2^, is increasing. It is affecting women more than men, and African American women (16.9%) more than Caucasian (9.3%), or Hispanic (8.9%) women (1). The number of bariatric surgeries performed yearly in the US has increased only minimally in the last few years and was estimated at 216,000 in 2016. Hence, only a small percentage of people meeting criteria for bariatric surgery, the most efficient and durable form of weight loss, actually benefit from it. Roux-en-Y gastric bypass (RYGB) was the dominant type of surgery performed in the US up to 2011. Vertical sleeve gastrectomy (VSG) is now the most performed surgery and represented 58% of all bariatric procedures in 2016 (2, 3). However, RYGB is the surgical model that has been studied the most to investigate gut mechanisms, independent of weight loss, that may contribute to post-operative glucose control. In addition, there are more long-term data on clinical remission of type 2 diabetes (T2D) after RYGB. Hence, this review will be more RYGB-centric.

The remarkable effect of bariatric surgery on T2D has generated considerable attention from the surgical, as well as the research community, in the last 12 years. Non-randomized observational studies have shown that bariatric surgery results not only in diabetes remission, but also decreases micro- and macro-vascular complications, cardiovascular disease risk and events, non-alcoholic steato-hepatitis (NASH) (4) and cancers (5–12). Cohort studies have shown increased longevity after bariatric surgery (10, 13). The effect of bariatric surgery on T2D remission is of particular interest. Both observational studies (14) and randomized controlled trials (RCTs) (15) show rates of remission varying from 15 to 100%, depending, in part, of the definition of diabetes remission (16, 17). Determinants of diabetes remission have been reviewed in meta-analysis (18) and the IDF-ADA Translational symposium (19). Pre-intervention β-cell function, use of insulin, known duration of T2D, HbA1C, age, surgery type, weight loss amount, genomics biomarkers, and the duration of follow-up after surgery remission, are all predictors of remission (20–25). The duration of follow up is certainly one of the key variables. In the Swedish Obesity Study (SOS), the rate of T2D remission decreases from 72%, at 2 years, to 36% at 10 years (5). Adams et al. show a decrease in the rate of remission from 75% at 2 years to 51% at 21 years in 84 patients with little attrition (90% follow up) (12). Arterburn et al. using electronic medical records, studied a large cohort of 4, 434 individuals with uncontrolled diabetes prior to surgery; of the 68.2% patients who initially remit their diabetes at 5 years, one third experience diabetes relapse 5–8 years after RYGB surgery (11). Overall, clinical parameters pre-intervention, surgery type, and post-surgery weight loss amount predict about 70% of remission rate. Predictive scores such as DiaRem (26) and ABCD (27) have been developed.

Pooling data from observational studies (14) and RTCs (15, 28–30), the rate of T2D remission is about 60% 2 years after RYGB. The mechanism by which RYGB results in this remarkable high rate of diabetes remission is not fully elucidated. The key question is whether diabetes remission is entirely weight loss dependent or not. If it is weight loss driven, then research should focus on the mechanisms, likely centrally mediated, by which patients eat less, lose about 30% of their total body weight and are able to keep the weight off, all goals unmatched with diet and exercise alone (31), or with pharmacotherapy (32). If some weight loss independent effects are at play in diabetes remission, they are likely gut-mediated. However, although RYGB results in many alterations of gut-mediated endocrine mechanisms, some of which play a role in post-prandial glucose control, their role in diabetes remission has not been fully demonstrated. The understanding of these mechanisms is crucial as it may help identify novel targets for the treatment of T2DM.

Calorie restriction with large (25–30%) and sustained (33–35) weight loss, are clearly important factors in the remission of diabetes after RYGB. They remove the chronic insult on the β-cell resulting from nutrient excess, i.e., glucose and lipid toxicity (36, 37), decrease inflammation (38–41), decrease fat mass and ectopic fat depots (42–44), and improve insulin sensitivity (29), all important modulators of metabolism. However, the benefit of the surgery on glucose control is apparent very rapidly, within days after RYGB surgery, prior to large amount of weight loss (45). In addition, the clinical observations that surgeries that alters the gastro intestinal track, such as RYGB, VSG, or biliopancreatic diversion (BPD), result in greater and more rapid diabetes remission than purely restrictive surgeries such as adjusted gastric banding (AGB), have prompted investigations of gastro-intestinal mediated mechanisms of glucose improvement. The regulation of blood glucose is complex and necessitates cross talk between the central nervous system, the endocrine pancreas, the liver, and the intestine (46). The intestine is the first line of contact with the environment, i.e., nutrient calorie load and composition, and plays a central role in post-prandial glucose control. The small intestine signals other organs via nutrient sensing, glucose transport, satiety and incretin hormones, bile acids metabolism, and the microbiome. Many of these intestinal pathways, reviewed below, contribute to glucose control, independent of weight loss, after RYGB (47).

The gut endocrine system regulates satiety and insulin secretion, both key factors in body weight and glucose control (48). Bariatric surgery alters the gut endocrine system in a favorable way to decrease appetite and improved glucose metabolism (49). After RYGB, ingested food empties rapidly from the small gastric pouch into the alimentary limb, and mix with the biliary and pancreatic exocrine secretion in the common limb (Figure 1). The rapid emptying of the reduced gastric pouch results in accelerated nutrient transit (50, 51) and alters the post-prandial gastro-intestinal hormonal chain of event. It enhances the release of satiety hormone such as cholecystokinin (CCK) (52, 53), peptide yy (PYY) (54), glucagon like peptide 1 (GLP-1) (55–57) and oxyntomodulin (58). A few clinical studies, using octreotide, demonstrated the role of gut peptides in increased satiety and decreased food reward after RYGB surgery (59, 60) (Figure 2). The release of the incretins GLP-1 (51, 61, 62), and of glucose dependent insulin peptide (GIP), in some (63–65) but not all (66) studies, is also enhanced by the accelerated transit; this improves the incretin effect on insulin secretion (55–57, 63, 67, 68) and lowers post-prandial glycemia (20). This exaggerated post-prandial release of GLP-1 occurs rapidly after the surgery (69), is independent of weight loss (51, 70) and can be entirely abolished by administration of the meal and/or glucose in the gastric remnant via a gastrostomy (67, 68). Although mean glucose levels improved after RYGB, the pattern of glucose levels during meals shows greater variability with earlier and higher glucose peaks, and lower post-prandial glycemia, at times in the hypoglycemic range, even if often asymptomatic. A small percentage of individuals experience debilitating neuroglycopenia after RYGB (71, 72), in relation to altered counter regulatory hormone response (73), increased insulin sensitivity (29) and decreased insulin clearance (74). The infusion of the GLP-1 receptor blocker exendin 9–39 prevents the large post-prandial insulin secretion and corrects the post-prandial neuroglycopenia; this illustrates the effect of endogenous GLP-1 on post-prandial glycemic control (75). The effect of exendin 9–39 on post-prandial glucose in individuals with normoglycemia, however, is more modest. Although exendin 9–39 can blunt post-prandial insulin secretion (76, 77), it results only in modest worsening of the glycemia (77–79).

**Figure 1:**
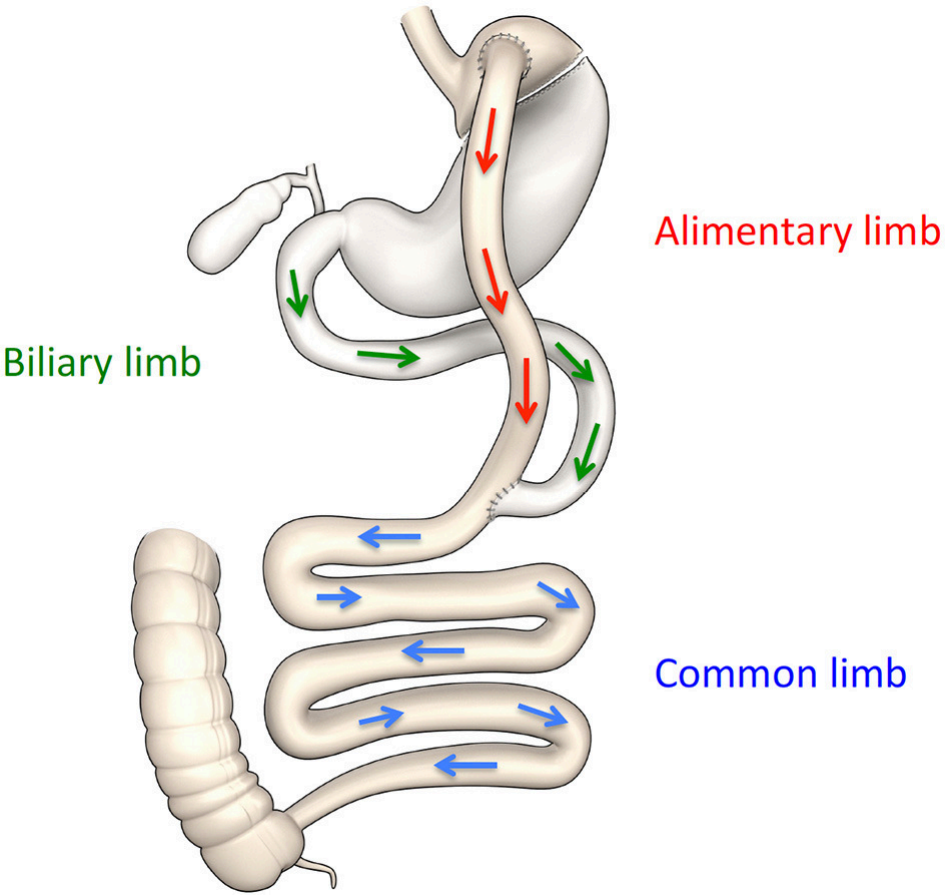
Schematic representation of anatomical changes after RYGB.

**Figure 2:**
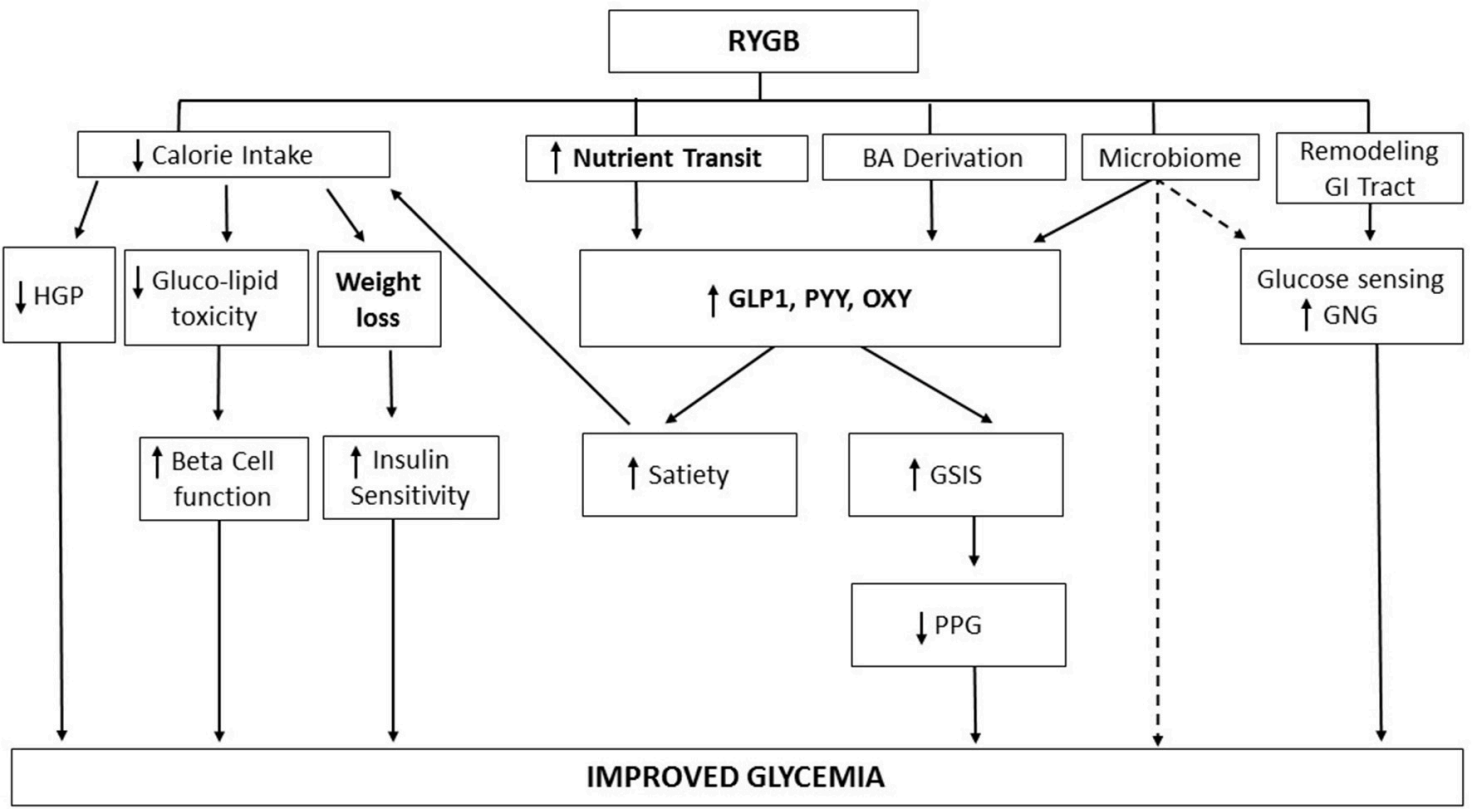
Mechanistic model of improved glycemia after RYGB. RYGB improves glucose metabolism via weight loss, and via weight-independent mechanisms, including stimulation of gut peptides, alteration of bile acids enterohepatic cycle, remodeling of the gastrointestinal track, and alteration of the microbiome. Solid lines: evidence based mechanisms; dashed lines: possible mechanisms. RYGB, Roux-en-Y gastric bypass surgery; HGP, hepatic glucose production; BA derivation, bile acids derivation; GI, gastro intestinal; GNG, gluconeogenesis; GLP-1, glucagon-like-peptide 1; PYY, peptide YY; OXY, oxyntomodulin; GSIS, glucose-stimulated insulin secretion; PPG, post-prandial glucose; ↑: increase; ↓: decrease.

The role of enhanced endogenous GLP-1 on the control of insulin secretion in response to oral glucose after RYGB is well demonstrated; its long-term implication on diabetes remission however remains elusive (79). Beta-cell function, assessed in response to intravenous glucose stimulus, improves only minimally and remains impaired in individuals in clinical diabetes remission and sustained weight loss, up to 3 years after RYGB (76). The reversal of post-prandial hyperinsulinemic hypoglycemia by the administration of food directly via gastrostomy in the remnant stomach, rather than *per os*, highlights the absence of permanent amelioration of the pancreatic endocrine function years after RYGB (80, 81). Therefore the increased meal-related insulin secretion after RYGB depends on enteric stimulation rather than on improved beta cells responsiveness to glucose (82) or to incretin stimuli (83). The persistent defect of beta cell function, overcome during meals, may explain, in part, the potential for diabetes relapse years after RYGB, in older patients who eat a less restrictive diet and regain some weight.

In addition to the intestinal endocrine function, other aspects of the gastro intestinal track play an important role in glucose control (84). The remodeling and reprogramming of the gastrointestinal track modifies intestinal glucose metabolism and glucose absorption and contributes to whole body metabolism after RYGB (85, 86). Interestingly this may not be the case after VSG (87) (Table 1). Troy et al. show increased intestinal gluconeogenesis after RYGB in mice, and effect abolished in GLUT-2 knockout mice (88). Others have shown an increased expression of genes involved in intestinal glucose transport and gluconeogenesis, in a rat bypass model (89–91), in association with decreased insulin resistance (92). Saeidi et al. demonstrated in rats that the active remodeling of the gastrointestinal tract increased intestinal cholesterol and glucose utilization, and contributed significantly to the improvement of whole body glucose metabolism after RYGB (93). Intestinal glucose transport is one of the key determinants of post-prandial glucose. The sodium-glucose transporter 1 (SGLT1) is responsible for the sodium-dependent, active uptake of glucose across the apical membrane of the small intestine (94). The expression of SGLT-1 increases after duodenal jenunal bypass (DJB) in rats (90) and after RYGB in humans (95). Baud et al. demonstrated, in a well characterized RYGB model in mini pigs (96), that the intestinal uptake of ingested glucose is blunted in the bile-deprived alimentary limb (Figure 1). Glucose absorption can be restored by the addition of either bile or sodium to the glucose meal, and is blocked with phlorizin. These studies provide direct evidence of a novel mechanism, via the reduction of active glucose-sodium transport, of decreased post-prandial glycemia after RYGB (96). More research is needed to understand the role of altered glucose transport, intestinal neoglucogenesis, and re-programming of the intestine on short and long-term glucose control after RYGB in humans, and its contribution to diabetes remission.

**Table 1:** Mechanisms of glycemic control after RYGB, VSG, and AGB.

	**RYGB**	**VSG**	**AGB**
Weight loss	+++	++	+
Accelerated nutrient transit	+	+	↔
↑ GLP-1, PYY, OXY	++	+/–	↔
Bile acid derivation	+	–	–
Circulating bile acid pool	↑	↑/↔	↓/↔
Remodeling GI tract	+	–	–
Microbiome	+	+	+

The change of bile acids metabolism after RYGB has been studied as potential mechanism of improved glucose control after bariatric surgery (97). Bile acids are synthesized by the liver, stored in the gallbladder and released in the duodenum in response to ingestion of nutrient. In non-operated individuals, after food intake, the chime, bile acids, and pancreatic exocrine secretions mix to enhance intestinal lipid digestion and absorption. After RYGB, in the absence of gastric fundus and pylorus, the ingested food empties rapidly from the gastric pouch; it then mix with bile acids and pancreatic secretions only in the common limb (Figure 1), precluding any duodenal absorption of nutrient (96). In addition to their role on lipid absorption, bile acids act as signaling molecules to regulate metabolism and inflammation (98). Bile acids are ligands of the nuclear receptor farnesoid X receptor (FXR) and the Takeda-G-protein- membrane receptor-5 TGR5 (99, 100), both receptors present in several organs that regulate metabolism. The role of the intestinal bile acids receptors as key regulators of glucose homeostasis was reviewed recently (101). The circulating bile acids concentrations (total molar sum) increase after RYGB in the fasted (102–106) and postprandial (77, 107–113) states in humans, as well as in rats and mini-pigs (114). The composition of the bile acids pool is also altered, and could contribute to the improvement of metabolism (106, 113). The rise of circulating bile acids after the surgery is delayed, occurs only a few months after the surgery and seems to be sustained overtime (106, 111, 113, 115). The underlying mechanisms of the elevated concentrations of circulating bile acids are unknown. Contrary to RYGB, calorie restriction and weight loss, either with (109) or without (112) AGB, decrease circulating bile acids concentrations (Table 1). Therefore, the rise in circulating bile acids after RYGB is not weight loss dependent. Experimental bile diversion, similarly to ileal transposition (116), are associated with increased circulating bile acids, increased postprandial GLP-1, weight loss and improved glucose tolerance (117–119). Possible explanations for the increased systemic pool of bile acids after RYGB are: increased hepatic synthesis and/or intestinal reabsorption, decreased fecal excretion and/or hepatic uptake or change in the microbiota. The increase in the peripheral but not in the portal circulation indicate that increase in bile acids systemic concentration after RYGB can be explained, in part, by a decrease of hepatic recapture, as shown after RYGB in mini pig (120). Whether the elevated systemic concentration of bile acids after RYGB in humans (113) is accompanied by increased concentration of luminal bile acids is unknown. One study in rats, show no change in luminal bile acids metabolism after RYGB and VSG (98, 121). Intestinal FXR is an important modulator of whole body metabolism. Pharmacological intestinal-specific activation of FXR reduces insulin resistance and stimulates adipose tissue browning, reduces lipids, inflammation, and atherosclerosis, while intestinal FXR inhibition favors non-alcoholic hepatic steatosis (NASH) (122). The effect of VSG on the improvement of glucose tolerance is reduced in FXR knock out (KO) mice (123). Bile acids, via activation of TGR5 signaling on the L cells, stimulate GLP-1 and participate in the control of glucose homeostasis (124–126). TGR5 seems to be required for the anti-hyperglycemic effect of VSG, as shown by two independent reports of VSG in TGR-5 KO mice (127, 128).

In all, results from clinical and animal studies suggest an important role of altered bile acids pool, composition, re-routing and signaling that may contribute to the metabolic effects of RYGB or VSG (Table 1). The elegant experiments of bile acids derivation and FXR and TGR5 KO propose a role for luminal bile acids in the improvement of metabolism after bariatric surgery. The clinical translation of these data, however, is still elusive. The temporal dissociation between the immediate rise of GLP-1 and the delayed increase in circulating bile acids, makes it less likely that the two processes are linked, at least in the early months after RYGB. Important information on intraluminal bile acids concentration after RYGB (or VSG) in humans is lacking. The composition, and therefore the function of the bile acids differs amongst species and add to the difficulty of translational research in this field. Finally, the large variability of the circulating concentrations of bile acids in humans studies (115) point out to other mechanisms, perhaps diet and/or microbiome dependent, that may modulate their composition and function.

Specific composition of the gut microbiome associates with pathological conditions such as cardiovascular disease, and with certain phenotypes like obesity and insulin resistance (129, 130). The link between gut microbiota composition and metabolic status is established through transplantation studies in humans and animals (131). However, the mechanism by which the gut microbiome maintains health or contributes to diseases is unknown. The change in microbiota composition, diversity and function is proposed as mechanism of some of the metabolic alterations after bariatric surgery (132–138). Transplantation of gut microbiota from RYGB mice (139) or patients (140) to germ-free mice reduces weight, fat mass, and induces metabolic improvements. Together, these studies indicate a possible link between gut flora modifications and metabolic changes after RYGB. Proposed mechanisms involve changes in glucose transport and sensing, GLP-1, short-chain fatty acids, lipogenesis, food intake, energy expenditure, adipose tissue metabolism, bile acids metabolism (141–146). One study however showed no link between alteration of the microbiome signature after RYGB and VSG and luminal metabolism of bile acids (121). The translational applicability of germ free mice experiments to humans is questionable. Human bariatric microbiota studies are often short term, lack controlled condition (diet, antibiotics and other drugs, metabolic status), favor description of composition rather than function of microbiome, and are based on feces, rather than luminal flora analysis. Future research will help identify whether the pre-surgery microbiome signature can be used to predict the metabolic response to the surgical intervention, and /or whether the change of microbiome composition and function can identify novel pathways of improved metabolism after various types of surgeries.

An important variable often overlooked in cross sectionals studies, is the change over time of many of the mechanisms described above. The accelerated nutrient transit time and stimulated GLP-1 release both occur immediately after RYGB and are sustained over time. However, the variance of the GLP-1 response increases between 1 month and 3 years post-surgery (147). We (113) and others (111) have demonstrated a temporal change of the pool of circulating bile acids after RYGB. Gut adaptation (hypertrophy, density of endocrine cells, glucose sensing, GNG) and the microbiome, are likely to undergo temporal transformation, in part, diet dependent. These data show the complexity of the gut physiology and adaptability, the difficulty of clinical studies, and the importance of longitudinal long-term studies for a better understanding of the contribution of the gut on post-prandial glycemia as well as diabetes remission.

In summary, RYGB results in T2DM remission as a result of large and sustained weight loss. RYGB also triggers weight independent gastro-intestinal mechanisms, including the stimulation of the incretins, the modulation of intestinal glucose transport and metabolism, the alteration of the entero-hepatic bile acids cycle, and change in the microbiome. These gut-related systems are inter-related as bile diversion impairs upper intestinal glucose uptake, nutrient malabsorption and bile acids can stimulate GLP-1, and the microbiome modulates many of these gastrointestinal targets. The mechanisms described above are likely to act in concert to contribute, with weight loss and calorie restriction, to glucose control after bariatric surgery (Figure 2). However, more clinical research needs to be done to understand the molecular mechanisms by which these different systems interact to improve glucose metabolism and to result in diabetes remission. The lack of normalization of beta cell function in response to IV glucose stimulus may be an important determinant of the future risk of diabetes relapse after RYGB surgery.

## Author contributions

All authors listed have made a substantial, direct and intellectual contribution to the work, and approved it for publication.

### Conflict of interest statement

The authors declare that the research was conducted in the absence of any commercial or financial relationships that could be construed as a potential conflict of interest.
